# Idiopathic Pulmonary Fibrosis in Elderly Patients: Analysis of the INSIGHTS-IPF Observational Study

**DOI:** 10.3389/fmed.2020.601279

**Published:** 2020-11-16

**Authors:** Gabriela Leuschner, Jens Klotsche, Michael Kreuter, Antje Prasse, Hubert Wirtz, David Pittrow, Marion Frankenberger, Jürgen Behr, Nikolaus Kneidinger, The INSIGHTS-IPF Registry Group, Stefan Andreas

**Affiliations:** ^1^Comprehensive Pneumology Center (CPC-M), Asklepios Klinik Gauting and Helmholtz Center Munich, Ludwig-Maximilians University, München, Germany; ^2^Department of Internal Medicine V, Ludwig-Maximilian University Munich, Munich, Germany; ^3^German Center for Lung Research, München, Germany; ^4^Epidemiology, German Rheumatism Research Center, A Leibniz Institute, Berlin, Germany; ^5^Center for Interstitial and Rare Lung Diseases, Pneumology, Thoraxklinik, University of Heidelberg, Heidelberg, Germany; ^6^Klinik für Pneumologie, Medizinische Hochschule Hannover, Hannover, Germany; ^7^Fraunhofer Institute for Toxicology and Experimental Medicine (ITEM), Hannover, Germany; ^8^Abteilung für Pneumologie, Department Innere Medizin, Neurologie und Dermatologie, Universitätsklinikum Leipzig AöR, Leipzig, Germany; ^9^Institut für Klinische Pharmakologie, Medizinische Fakultät, Technische Universität Dresden, Dresden, Germany

**Keywords:** aging, elderly, antifibrotic therapy, prognosis, multivariate analysis

## Abstract

**Background:** An association between idiopathic pulmonary fibrosis (IPF) and advancing age is suspected since IPF occurs primarily in patients over 60 years of age. Though, little is known about the disease in the elderly. The aim of this study was to characterize elderly IPF patients using data from the longitudinal, German-wide INSIGHTS-IPF registry.

**Methods:** Patients were grouped into elderly (≥75 years) and nonelderly IPF (<75 years) at the time of enrollment into the study. Baseline clinical characteristics, comorbidities, health related quality of life (HRQoL), medical therapy and survival were compared between age groups. Effects of antifibrotic therapy on forced vital capacity (FVC) were analyzed over 24 months.

**Results:** Of 1,009 patients, 350 (34.7%) were ≥75 years old. Elderly IPF patients compared to younger patients had a higher number of comorbidities (3.6 ± 2.5 vs. 2.8 ± 2.3; *p* < 0.001). The mean ± SD EQ-5D score (0.64 ± 0.21 vs. 0.69 ± 0.21; *p* = 0.005), and the overall WHO-5 score (13.1 ± 5.9 vs. 14.3 ± 6.0; *p* = 0.015) were significantly lower while the UCSD-SOBQ (52.6 ± 31.2 vs. 45.5 ± 31.2; *p* = 0.030) was significantly higher in elderly patients, indicating a more impaired HRQoL and more breathlessness. At baseline, 55.4% of elderly and 56.8% of nonelderly patients with IPF were treated with antifibrotic therapy (*p* = 0.687). For FVC decline after initiation of antifibrotic therapy, there was neither a significant difference between age groups at the different time points over 24 months (beta: 0.41; 95%-CI: −0.98 to 1.81; *p* = 0.563) nor over the whole course of time (beta: −0.05; 95%-CI: −0.20 to 0.09; *p* = 0.478). All-cause mortality was higher in elderly patients (49.1 vs. 37.9%; HR 1.65; 95%-CI 1.36–2.00; *p* < 0.001). Antifibrotic therapy was associated with improved survival in IPF patients, independent from age (<75 years: beta 0.76; 95%-CI: 0.59–0.99; *p* = 0.049; ≥75 years: beta 0.71; 95%-CI: 0.51–0.98; *p* = 0.043).

**Conclusion:** In real life, a significant proportion of IPF patients are ≥75 years old, characterized by higher number of comorbidities and global reduced HRQoL. However, the effect of an antifibrotic therapy was similar between age groups and associated with a survival benefit emphasizing the importance for an early antifibrotic therapy in IPF, independent from age.

## Introduction

Idiopathic pulmonary fibrosis (IPF) is a chronic interstitial lung disease (ILD) characterized by clinical symptoms such as dyspnea, cough and increasing immobility (1). IPF occurs primarily in patients over 60 years of age (1, 2), for which reason a connection with aging processes has been suspected. The aging lung is subject to biological changes which make it more susceptible to disease. Recently, genetic alterations have been identified which are associated with an increased risk for the development of IPF, but may also increase the risk of other lung diseases associated with aging such as chronic obstructive pulmonary disease (COPD) or lung cancer (3). Although the exact relationship between aging and the pathogenesis of IPF is still unclear, there are a number of processes, which can be found in the disease including telomerase shortening, cell aging, mitochondrial dysfunction, and dysregulation of the extra-cellular matrix (4, 5).

Additionally, the advanced age of patients with IPF in general is associated with a higher number of comorbidities and reduced quality of life. Indeed, studies have shown that patients with IPF often suffer from comorbidities (6, 7), which may also be due to advanced age.

IPF is a disease with dismal prognosis. For only a few years now, there are two antifibrotic drugs approved that, while they cannot cure the disease, can slow down the loss of forced vital capacity (FVC). A subgroup analysis of the INPULSIS trials detected no differences between patients below and above the age of 65 years in the primary endpoint (annual rate of decline in FVC) and key secondary endpoints [change from baseline in St. George's Respiratory Questionnaire (SGRQ) total score or time to first acute exacerbation] in patients treated with nintedanib vs. placebo (8). However, it is unclear if antifibrotic therapy is consistently initiated and effective in IPF patients over the age of 75 years, and if the effect also can be reproduced outside randomized controlled trials, under real-life conditions.

The aim of our study was to characterize elderly patients with IPF (≥75 years) in comparison to younger IPF patients with respect to comorbidities and quality of life. Further, we wanted to evaluate the use of antifibrotic therapy and its effect on lung function and survival in elderly compared to younger patients with IPF.

## Methods

### Study Population

The INSIGHTS-IPF (“Investigating significant health trends in idiopathic pulmonary fibrosis”) is a German, nationwide, investigator-initiated cohort study (registered at Clinicaltrials.gov NCT01695408). Since November 2012, patients with IPF have continuously been enrolled in routine clinical care in 19 pulmonary specialist centers in Germany. Patients are eligible if they are ≥18 years old, have a study-site diagnosis of IPF following the 2011 international IPF guideline and have provided written informed consent (9). The study was approved by the Ethics Committee of the Medical faculty, Technical University, Dresden, Germany in 2012, and by additional local ethics committees in accordance with local requirements. The methodology and structure of the registry as well as a detailed description of the baseline characteristics have been reported previously (9–11). The ongoing INSIGHTS-IPF study has no explicit exclusion criteria. Clinical data are collected at the initial enrollment, and thereafter every 6–12 months. Clinical events like hospitalization, acute exacerbation (as judged by the treating physician) and death are recorded at these follow-up visits. For the data collection an internet-based, secure case report form (eCRF) was used. For the present analysis, all patients from the INSIGHTS-IPF registry with at least one follow-up assessment were included. The last patient of our analysis was included on December 11, 2019. Data cut-off date was on April 29, 2020. Based on the age at enrollment patients were grouped into “elderly IPF” (≥75 years) and “nonelderly IPF” (<75 years).

### Clinical Data

At baseline and the study visits (every 6–12 months), routine pulmonary function tests were performed and different measures were documented, including FVC % predicted, diffusing capacity of the lung for carbon monoxide (DLCO) % predicted, the forced expiratory volume in 1 s (FEV1), and six-minute walk distance (6MWD). Based on lung function, age and gender, the Gender, Age, Physiology (GAP) index was calculated (12). Based on clinical judgement of overall disease course, when possible, the treating physician categorized the patient as stable disease, slow progression, rapid progression (10).

A range of comorbidities was documented and recorded at baseline. Based on these pre-selected comorbidities, the individual number of comorbidities was calculated.

Specific therapy, including formerly used immunosuppressants, anticoagulation, antifibrotic therapy, and long-term oxygen were documented at baseline and thereafter during study visits. For the follow-up analysis of FVC decline under antifibrotic therapy only patients, in whom an antifibrotic therapy was newly started, were eligible.

### HRQoL

To evaluate health-related quality of life (HRQoL) the St. George's Respiratory Questionnaire (SGRQ) and the University of California San Diego Shortness of Breath Questionnaire (UCSD SOB) were applied as described in detail before (13, 14). In addition, the World Health Organization-5 Well-Being Index (WHO-5) was used. Based on 5 items, the WHO-5 is a short questionnaire evaluating well-being. The total score ranges from 0 to 25, whereby lower scores correspond to a lower level of well-being. A score below 13 can indicate a possible depression. Further, the EuroQol five-dimensional questionnaire (EQ-5D), generated by the EuroQol group, is used for evaluation of general healthcare and cost-utility (15). It consists of five domains (mobility, self-care, usual activities, pain or discomfort, and anxiety or depression) and a visual analog scale (VAS). A sum utility score can be calculated based on the five domain scores, which ranges from 0 to 1 (perfect health state). The scores of the VAS range from 0 (health state equivalent to death) to 100 (best imageable health state).

### Data Analysis

Continuous variables are presented as the mean ± standard deviation (SD), and categorical variables are summarized by frequency and percentage. Continuously distributed sociodemographic and clinical parameters were compared by a *t*-test between nonelderly and elderly patients. A Chi-square test was used to compare categorical parameters between the two groups. The risk of mortality was investigated by a multivariable Cox proportional hazard model for overall mortality in the total sample and separately in nonelderly and elderly patients. For survival analysis, patients who underwent lung transplantation were censored at the time of lung transplantation. Potential predictor variables for overall mortality were assessed in univariable analyses. All parameters with a significance level of *p* < 0.10 were included in the multivariable model. All treatment episodes with antifibrotic therapy (nintedanib or pirfenidone) were identified at enrollment and during follow-up in the registry. There was no pre-defined treatment threshold for initiation of an antifibrotic therapy. Patients with a maximum visit of 20 days before and 20 days after the start of antifibrotic therapy were selected for the analyses of the course of FVC % pred. after start of antifibrotic therapy as described previously (16). Since the date of the start of an antifibrotic therapy did not necessarily correspond exactly to the study visit date, the lung function values at the start of antifibrotic therapy were extracted from the visits +/– 20 days before the start of therapy. The change in FVC % pred. up to 24 months after therapy start was analyzed by a generalized linear mixed model. Data management and statistical analyses were conducted with use of STATA 12.1 (StataCorp LP. Stata Statistical Software: Release 12. College Station, TX, USA).

## Results

### Patient Characteristics

Of 1,009 patients enrolled in the INSIGHTS IPF registry until December 2019, 350 (34.7%) were ≥75 years of age. The most common initial symptoms in elderly and nonelderly patients with IPF were dyspnea (86.3 and 85.7%; *p* = 0.811) and cough (68.3 and 72.8%; *p* = 0.128). Bibasilar crackles were found in 80.0% of elderly and 82.4% of nonelderly patients (*p* = 0.350) on auscultation. Baseline characteristics of elderly and nonelderly IPF patients are shown in Table 1. There were marked differences with regard to mean age at enrollment (78.6 ± 3.1 years vs. 65.4 ± 7.4 years; *p* < 0.001), age at symptoms onset (75.4 ± 5.1 years vs. 61.6 ± 8.8 years; *p* < 0.001), age at IPF diagnosis (76.8 ± 4.7 years vs. 63.5 ± 8.3 years; *p* < 0.001) and duration since first IPF symptoms (3.1 ± 3.7 years vs. 3.8 ± 4.3 years; *p* < 0.026) between elderly and nonelderly IPF patients. At baseline, elderly IPF patients had significantly higher FVC (70.6 ± 17.4% pred. vs. 66.2 ± 18.8% pred.; *p* < 0.001) and FEV1 (79.9 ± 18.5% pred. vs. 73.6 ± 19.4% pred.; *p* < 0.001) and lower DLCo (34.3 ± 14.0% pred. vs. 36.8 ± 17.1% pred.; *p* = 0.030) or PaO2 (67.1 ± 10.7 mmHg vs. 69.3 ± 11.7 mmHg; *p* = 0.010), respectively. The mean ± SD BMI of elderly IPF was lower (26.7 ± 3.9 vs. 27.9 ± 4.3; *p* < 0.001) with less patients suffering from obesity (18.6 vs. 27.3%; *p* < 0.001).

**Table 1:** Baseline characteristics of elderly and nonelderly patients with IPF.

		**Age group <75 *n* = 659**	**Age group ≥75 years *n* = 350**	***p*-value**
Sex, *n* (%)				
	Male	534 (81.0%)	280 (80.0%)	0.693
	Female	125 (19.0%)	70 (20.0%)	
Age in years, mean (sd)		65.4 (7.4)	78.6 (3.1)	<0.001
BMI in kg/m^2^, mean (sd)		27.9 (4.3)	26.7 (3.9)	<0.001
	Underweight	5 (0.8%)	6 (1.7%)	<0.001
	Normal weight	148 (22.5%)	109 (31.1%)	
	Overweight	326 (49.5%)	170 (48.6%)	
	Obesity	180 (27.3%)	65 (18.6%)	
Smoking status				
	Never	221 (33.5%)	131 (37.4%)	0.176
	Former stopped	421 (63.9%)	215 (61.4%)	
	Current	17 (2.6%)	4 (1.1%)	
Age at symptom onset, mean (sd)		61.6 (8.8)	75.4 (5.1)	<0.001
Duration since first IPF symptoms				
	In years, mean (sd)	3.8 (4.3)	3.1 (3.7)	0.026
Age at IPF diagnosis, mean (sd)		63.5 (8.3)	76.8 (4.7)	<0.001
Duration since IPF diagnosis				
	In months, mean (sd)	23.1 (33.2)	21.6 (41.3)	0.537
	In years, mean (sd)	1.9 (2.8)	1.8 (3.5)	0.537
	<3 months	200 (31.2%)	114 (33.3%)	0.183
	3 to <6 months	61 (9.5%)	43 (12.6%)	
	6+ months	381 (59.4%)	185 (54.1%)	
IPF diagnosis was based on				
	HRCT	569 (86.3%)	319 (91.1%)	0.025
	Surgical lung biopsy/histology	272 (41.3%)	70 (20.0%)	<0.001
Current NYHA class				0.248
	I	41 (14.2%)	11 (8.1%)	
	II	120 (41.5%)	54 (39.7%)	
	III	117 (40.5%)	65 (47.8%)	
	IV	11 (3.8%)	6 (4.4%)	
Current Borg dyspnea index, mean (sd)		2.2 (2.3)	2.1 (2.2)	0.493
6-min walk distance, mean (sd)		299.3 (196.5)	275.5 (164.6)	0.063
Lung function test, mean (sd)				
	FEV1	73.6 (19.4)	79.9 (18.5)	<0.001
	FVC	66.2 (18.8)	70.6 (17.4)	<0.001
	TLC	70.2 (20.2)	69.4 (14.6)	0.522
	DLCO	36.8 (17.1)	34.3 (14.0)	0.030
	PaO2	69.3 (11.7)	67.1 (10.7)	0.010
Physician's global assessment of clinical course of IPF				
	Stable disease	268 (40.7%)	150 (42.9%)	0.189
	Slow progression	173 (26.3%)	85 (24.3%)	
	Rapid progression	62 (9.4%)	21 (6.0%)	
	No judgement possible	156 (23.7%)	94 (26.9%)	

*Data are presented as mean ± SD, or n (%)*.

*BMI, body mass index; IPF, idiopathic pulmonary fibrosis; HRCT, high resolution computed tomography; NYHA, New York Heart Association; FEV1, forced expiratory volume in 1; FVC, forced vital capacity; TLC, total lung capacity; DLCO, diffusing capacity of the lung for carbon monoxide*.

UIP pattern was found in 64.3% of elderly and in 60.0% of nonelderly IPF patients. A possible UIP pattern was present in 25.7% of elderly and 29.6% of nonelderly patients and a HRCT inconsistent with UIP was found in 1.1% of elderly and 1.1% of nonelderly patients. In 8.9% of elderly and 9.0% of nonelderly patients no information on the HRCT pattern was provided. The diagnosis of IPF was more often based on HRCT (91.1 vs. 86.3%; *p* = 0.025) in elderly IPF and less patients had undergone surgical lung biopsy/histology (20.0 vs. 41.3%; *p* < 0.001). In elderly patients, there were no difference between patients without surgical lung biopsy and patients with surgical lung biopsy in terms of FVC (70.5 ± 17.7% pred. vs. 71.1 ± 16.2% pred.; *p* = 0.821), DLCO (33.7 ± 14.2% pred. vs. 37.3% pred.; *p* = 0.105), PaO2 (66.8 ± 10.6 mmHg vs. 68.6 ± 10.6 mmHg; *p* = 0.302), number of comorbidities (3.6 ± 2.5 vs. 3.5 ± 2.3; *p* = 0.755), and HRQoL (EQ-5D score: 66.2 ± 24.8 vs. 63.6 ± 20.6; *p* = 0.469; WHO-5: 13.0 ± 5.9 vs. 13.8 ± 6.3; *p* = 0.398; SGRQ: 57.8 ± 20.8 vs. 56.3 ± 26.1; *p* = 0.704). Diagnosis of IPF was based on multidisciplinary discussion in 63.4% of elderly patients and 62.7% of nonelderly patients, while in 15.7% of elderly and 15.8% of nonelderly the diagnosis of IPF was not based on a multidisciplinary discussion. There was no data available if or not a multidisciplinary discussion was performed in 20.9% of elderly and 21.6% of nonelderly IPF.

Further, elderly IPF patients had a significantly higher number of comorbidities at baseline (3.6 ± 2.5 vs. 2.8 ± 2.3; *p* < 0.001; Table 2). Comorbidities that were more prevalent in elderly IPF included left heart failure (10.3 vs. 4.7%; *p* = 0.001), coronary artery disease (36.9 vs. 22.5%; *p* < 0.001), peripheral arterial disease (5.4 vs. 2.6%; *p* = 0.020), atrial fibrillation (15.4 vs. 5.6%; *p* < 0.001), and arterial hypertension (63.4 vs. 50.4%; *p* < 0.001). In contrast, depression and depressive disorder (2.6 vs. 7.3%; *p* = 0.002) and anxiety (1.7 vs. 4.9%; *p* = 0.013) were more often seen in nonelderly IPF. At baseline, lung cancer was present in five (1.4%) elderly and eleven (1.7%) nonelderly patients (*p* = 0.771). During the follow-up, two (0.6%) elderly and five (0.8%) nonelderly patients were newly diagnosed with lung cancer (*p* = 0.773).

**Table 2:** Comorbidities and number of comorbidities in elderly and nonelderly patients with IPF.

	**Age group <75 years *n* = 659**	**Age group ≥75 years *n* = 350**	***p*-value**
Left heart disease, *n* (%)	31 (4.7)	36 (10.3)	0.001
Coronary artery disease, *n* (%)	148 (22.5)	129 (36.9)	<0.001
Cerebrovascular disease^a^, *n* (%)	45 (6.8)	28 (8.0)	0.494
Peripheral arterial disease^b^, *n* (%)	17 (2.6)	19 (5.4)	0.020
Atrial fibrillation, *n* (%)	37 (5.6)	54 (15.4)	<0.001
Deep venous thrombosis, *n* (%)	13 (2.0)	10 (2.9)	0.370
Pulmonary arterial embolism, *n* (%)	10 (1.5)	11 (3.1)	0.082
Pulmonary hypertension, *n* (%)	96 (14.6)	59 (16.9)	0.337
Arterial hypertension, *n* (%)	332 (50.4)	222 (63.4)	<0.001
Diabetes mellitus, *n* (%)	140 (21.2)	92 (26.3)	0.070
Emphysema, *n* (%)	65 (9.9)	36 (10.3)	0.832
Lung cancer, *n* (%)	11 (1.7)	5 (1.4)	0.771
Obstructive sleep apnoea, *n* (%)	73 (11.1)	29 (8.3)	0.161
Depression/depressive disorder, *n* (%)	48 (7.3)	9 (2.6)	0.002
Anxiety, *n* (%)	32 (4.9)	6 (1.7)	0.013
Other comorbid diseases, *n* (%)	338 (51.3)	189 (54.0)	0.412
Number of comorbidities, mean (sd)	2.8 (2.3)	3.6 (2.5)	<0.001
None, *n* (%)	89 (13.5)	29 (8.3)	0.001
1–3, *n* (%)	354 (53.7)	165 (47.1)	
≥4, *n* (%)	216 (32.8)	156 (44.6)	

*Data are presented as mean ± SD, or n (%)*.

a *Carotid stenosis, stroke*.

b *Symptomatic or ankle brachial index <0.8*.

Medical therapy at baseline is shown in Table 3. Elderly IPF patients were more often on prophylactic (16.3 vs. 7.8%; *p* < 0.001) or therapeutic anticoagulation (18.9 vs. 9.6%; *p* < 0.001) in comparison to nonelderly IPF.

**Table 3:** Medical therapy at baseline in elderly and nonelderly patients with IPF.

	**Age group <75 years *n* = 659**	**Age group ≥75 years *n* = 350**	***p*-value**
Prednisone, *n* (%)	136 (20.6)	64 (18.3)	0.372
Other steroid, *n* (%)	9 (1.4)	8 (2.3)	0.280
Azathioprine, *n* (%)	11 (1.7)	3 (0.9)	0.294
Cyclophosphamide, *n* (%)	1 (0.2)	0 (0.0)	0.466
Mycophenolate mofetil, *n* (%)	1 (0.2)	0 (0.0)	0.466
N-Acetylcysteine, *n* (%)	144 (21.9)	69 (19.7)	0.429
Other, *n* (%)	16 (2.4)	9 (2.6)	0.889
Anticoagulation–prophylactic, *n* (%)	51 (7.8)	57 (16.3)	<0.001
Anticoagulation–therapeutic, *n* (%)	63 (9.6)	66 (18.9)	<0.001
Pirfenidone, *n* (%)	256 (38.9)	112 (32.0)	0.032
Nintedanib, *n* (%)	119 (18.1)	82 (23.4)	0.042
Antifibrotic therapy (nintedanib or pirfenidone), *n* (%)	374 (56.8)	194 (55.4)	0.687
Long-term oxygen therapy, *n* (%)	200 (30.4)	109 (31.1)	0.795

*Data are presented as n (%)*.

HRQoL was assessed using different standardized questionnaires (Table 4), which were available in 752 patients. The EQ-5D score (0.64 ± 0.21 vs. 0.69 ± 0.21; *p* = 0.005), EQ-5D VAS (56.9 ± 19.4 vs. 61.6 ± 19.8; *p* = 0.002), the overall WHO-5 score (13.1 ± 5.9 vs. 14.3 ± 6.0; *p* = 0.015), and the number of patients with WHO-5 scores <13 (54.9 vs. 43.0%; *p* = 0.003) showed significantly reduced HRQoL in elderly patients in comparison to nonelderly IPF. Breathlessness was more commonly reported by elderly patients (mean ± SD UCSD-SOBQ 52.6 ± 31.2 vs. 45.5 ± 31.2; *p* = 0.030). The overall assessment of current health status as well as the overall SGRQ and its subdomains symptoms, activity and impacts did not show significant differences between elderly and nonelderly patients.

**Table 4:** Health-related quality of life in IPF.

		**Age group <75 years *n* = 659**	**Age group ≥75 years *n* = 350**	***p*-value**
Overall assessment of current health state, *n* (%)				0.067
	Very good	6 (1.2)	1 (0.4)	
	Good	144 (28.5)	48 (19.7)	
	Medium	257 (50.8)	135 (55.3)	
	Poor	89 (17.6)	55 (22.5)	
	Very poor	10 (2.0)	5 (2.1)	
EQ-5D, mean (sd)				
	VAS 0–100	61.6 (19.8)	56.9 (19.4)	0.002
	Score	0.69 (0.21)	0.64 (0.21)	0.005
WHO-5, mean (sd)		14.3 (6.0)	13.1 (5.9)	0.015
	WHO-5 score <13	209 (43.0%)	130 (54.9%)	0.003
SGRQ, mean (sd)				
	SGRQ	47.2 (20.8)	49.4 (20.1)	0.172
	SGRQ symptoms	56.8 (20.9)	57.5 (21.8)	0.665
	SGRQ activity	60.7 (24.7)	64.3 (22.9)	0.062
	SGRQ impacts	37.2 (22.2)	37.4 (20.7)	0.897
UCSD Shortness of breath, mean (sd)		45.5 (31.2)	52.6 (31.2)	0.030

*Data are presented as mean ± SD, or n (%)*.

*EQ-5D, EuroQol five-dimensional questionnaire; WHO-5, World Health Organization-5 Well-Being Index; SGRQ, St. George's Respiratory Questionnaire; USCD-SOBQ, University of California San Diego Shortness of Breath Questionnaire*.

### Antifibrotic Therapy

At baseline, 55.4% (*n* = 194) of elderly patients and 56.8% (*n* = 374) of nonelderly patients were on antifibrotic therapy, with more elderly patients on nintedanib (23.4 vs. 18.1%; *p* = 0.042) but more nonelderly patients treated with pirfenidone (32.0 vs. 38.9%; *p* = 0.032; see Table 3). Of the 568 patients with antifibrotic therapy at baseline, 20.1% elderly and 20.9% nonelderly patients discontinued antifibrotic therapy during the follow-up (*p* = 0.868). While significantly more elderly had to discontinue antifibrotic therapy due to lack of tolerability (79.5 vs. 52.6%; *p* = 0.019), reasons for discontinuation of antifibrotic therapy were less often due to efficacy failure (15.4 vs. 34.6%) and other reasons (5.1 vs. 12.8%) compared to nonelderly patients. Dose distribution of pirfenidone and nintedanib did also not differ significantly between elderly and nonelderly IPF. Pirfenidone was taken in full dose (2,403 mg/day) by 61.7% of elderly patients who took pirfenidone and 67.2% of nonelderly patients (*p* = 0.352) who took pirfenidone. The full dose 300 mg/day nintedanib were taken by 80.5% elderly patients who took nintedanib and 83.5% nonelderly patients (*p* = 0.537) who took nintedanib.

**Figure 1:**
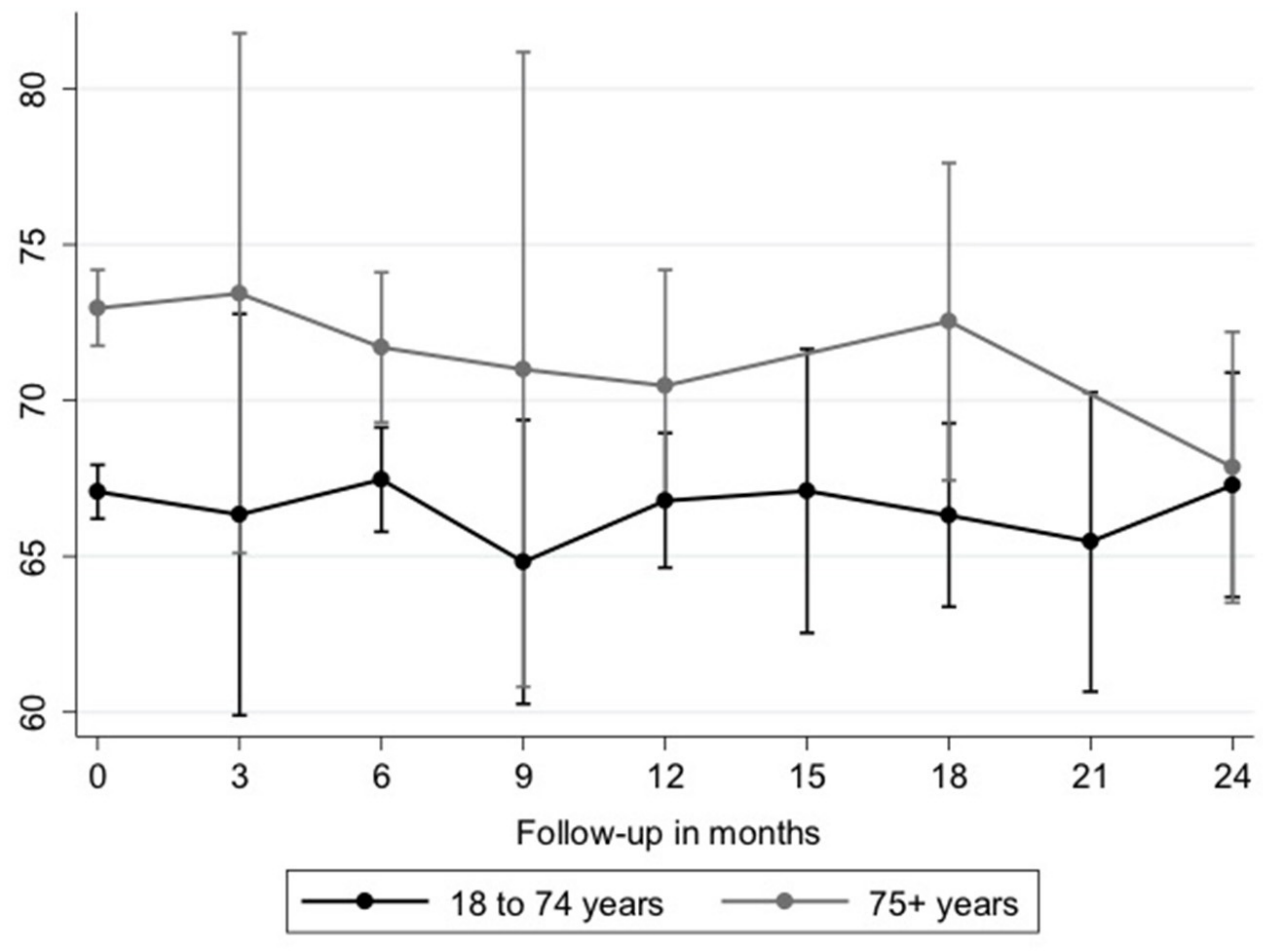
Longitudinal course of FVC (% pred.). FVC decline is shown in elderly (gray line, *n* = 148) and nonelderly (black line, *n* = 294) patients with IPF. Differences were not statistically significant. FVC, forced vital capacity; IPF, idiopathic pulmonary fibrosis.

Follow-up data after initiation of antifibrotic therapy was available in 148 elderly (42.3%) and 294 nonelderly (44.6%) patients. In elderly patients, FVC% pred. was significantly higher in comparison to nonelderly patients at the time of initiation of antifibrotic therapy [71.7% (95%-CI: 68.1–75.2) vs. 66.5 (95% CI: 64.1–68.9) *p* = 0.002]. Using a model analyzing FVC decline after initiation of antifibrotic therapy in patients with IPF, on average, there was no significant difference between age groups at any time point over 24 months (beta: 0.41; 95%-CI: −0.98 to 1.81; *p* = 0.563; Table 5 and Figure 1). Additionally, over the course of time, there was no significant difference between elderly and nonelderly patients (beta: −0.05; 95%-CI: −0.20 to 0.09; *p* = 0.478; Table 5). Other associations between FVC decline under antifibrotic therapy and different clinical variables are shown in detail in Table 5.

**Table 5:** Multivariable model of FVC decline after initiation of antifibrotic therapy in patients with IPF.

		**Beta**	**95%CI**	***p*-value**
Time		−0.18	−0.26 to −0.10	<0.001
Age group 75+ years		0.41	−0.98 to 1.81	0.563
Time × Age group 75+ years		−0.05	−0.20 to 0.09	0.478
Female		−0.37	−1.69 to 0.95	0.581
Death during Follow-up		−1.90	−2.93 to −0.86	<0.001
FVC at treatment start		0.94	0.91–0.97	<0.001
6MWD		0.00	0.00–0.01	0.041
Physician's global assessment of clinical course of IPF				
	Slow progression	−2.83	−4.11 to −1.54	<0.001
	Rapid progression	−3.23	−5.69 to −0.78	0.010
Number of comorbidities		0.23	0.00–0.46	0.046

*FVC, forced vital capacity; 6MWD, 6 minutes walking distance; IPF, idiopathic pulmonary fibrosis*.

### Survival

**Figure 2:**
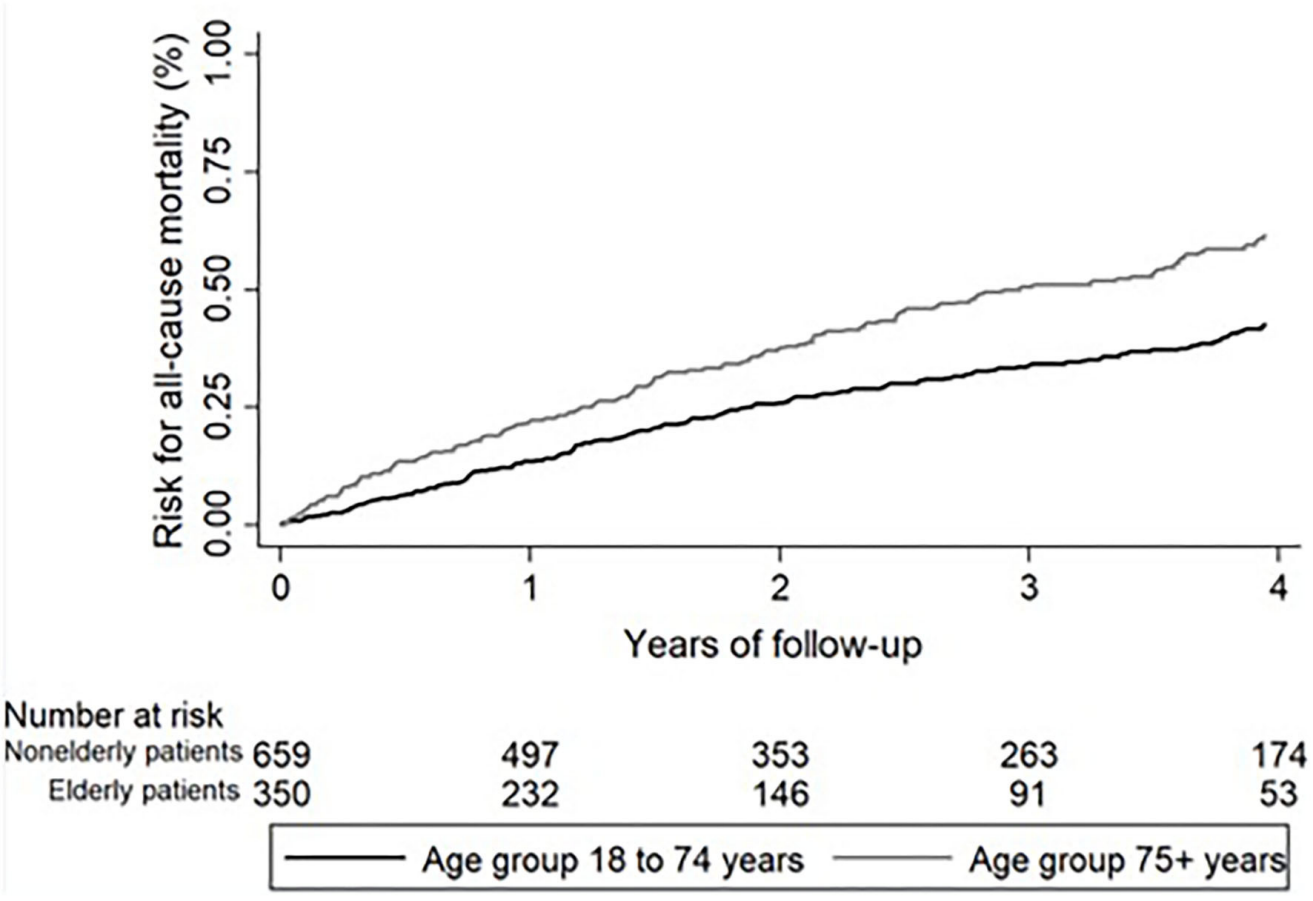
All-cause mortality over 4 years in patients with IPF. All-cause mortality was significantly higher in elderly (black line) compared to nonelderly patients with IPF (gray line). IPF, idiopathic pulmonary fibrosis.

In elderly IPF patients, the mean ± SD follow-up was 1.9 ± 1.3 years (median 1.5; IQR: 0.7–3.0) and in nonelderly patients 2.3 ± 1.4 years (median 2.1; IQR: 1.0–4.0). During the follow-up, 172 (49.1%) elderly and 250 (37.9%) nonelderly patients died. Reasons for death are shown in detail in Table 6. The all-cause mortality was higher in elderly IPF (49.1 vs. 37.9%; HR 1.65; 95%-CI 1.36–2.00; *p* < 0.001; Figure 2). While all other reasons for death did not show significant differences between elderly and nonelderly IPF, more elderly patients died due to unknown reasons for death (26.0 vs. 15.0%; HR 2.23; 95%-CI: 1.68–2.96; *p* < 0.001). During the follow-up one (0.3%) elderly patients with IPF and three (0.5%) nonelderly patients died due to lung cancer (0.90; 95%-CI: 0.11–7.66; *p* = 0.926).

**Table 6:** Reasons for death in elderly and nonelderly patients with IPF.

	**Age group <75 years *n* = 659**	**Age group ≥75 years *n* = 350**	**HR**	**95%CI**	***p*-value**
All cause mortality	250 (37.9%)	172 (49.1%)	1,65	1.36–2.00	<0.001
Death related to IPF	105 (15.9%)	57 (16.3%)	1,28	0.93–1.77	0,131
Death by respiratory failure	54 (8.2%)	25 (7.1%)	1,06	0.66–1.71	0,809
Death by acute exacerbation	20 (3.0%)	7 (2.0%)	0,77	0.32–1.86	0,566
Death by right heart failure	4 (0.6%)	1 (0.3%)	0,70	0.07–6.71	0,754
Death by respiratory infection/pneumonia	18 (2.7%)	14 (4.0%)	1,81	0.89–3.70	0,102
Death related to IPF but unknown reason	29 (4.4%)	17 (4.9%)	1,44	0.79–2.63	0,230
Death by complicating comorbidity	22 (3.3%)	9 (2.6%)	0,96	0.44–2.09	0,921
Death by other cause not related to IPF	24 (3.6%)	15 (4.3%)	1,51	0.80–2.86	0,201
Reasons for death unknown	99 (15.0%)	91 (26.0%)	2,23	1.68–2.96	<0.001
Lung transplantation	42 (6,4%)	0 (0.0%)			
Combined endpoint (all cause mortality/lung transplantation)	283 (42.9%)	172 (49.1%)	1,47	1.22–1.78	<0.001

*IPF, idiopathic pulmonary fibrosis*.

For the mortality analysis, patients who underwent lung transplantation were censored at the time of lung transplantation [elderly: *n* = 0 (0.0%); nonelderly: *n* = 42 (6.4)]. Choosing a combined endpoint of all-cause mortality and lung transplantation also resulted in a higher transplant-free mortality in elderly IPF (49.1 vs. 42.9%; HR 1.47; 95%-CI 1.22–1.78; *p* < 0.001).

Multivariable analysis for all-cause mortality identified the age group ≥75 years (HR 1.49; 95%-CI: 1.20–1.85; *p* < 0.001), FVC (HR 0.98; 95%-CI: 0.98–0.99; *p* < 0.001), GAP stage II (HR 1.91; 95-CI: 1.34–2.74; *p* < 0.001) and III (HR 1.76; 95%-CI: 1.22–2.55; *p* < 0.01), the categorization into slow (HR 1.38; 95%-CI: 1.08–1.76; *p* = 0.009) and rapid progressive (HR 1.76; 95%-CI: 1.22–2.55; *p* = 0.002) and no antifibrotic therapy (HR 1.35; 95%-CI: 1.10–1.65; *p* < 0.01) as being significant predictors for mortality in the whole study population (Table 7). Antifibrotic therapy was associated with a significantly better survival independent from age groups (<75 years: beta 0.76; 95%-CI: 0.59–0.99; *p* = 0.049; ≥75 years: beta 0.71; 95%-CI: 0.51–0.98; *p* = 0.043).

**Table 7:** Multivariable model assessing the effect on survival in patients with IPF.

		**Total (*****n*** **=** **1,009)**			**Age group** **<75 years (*****n*** **=** **659)**			**Age group** **≥75 years (*****n*** **=** **350)**		
		**HR**	**95%CI**	***p*-value**	**HR**	**95%CI**	***p*-value**	**HR**	**95%CI**	***p*-value**
Age group 75+ years		1.49	1.20–1.85	<0.001						
Female		1.04	0.81–1.33	0.752	1.12	0.83–1.51	0.475	0.84	0.55–1.28	0.416
Duration since first IPF symptoms (years)		0.98	0.95–1.00	0.088	0.99	0.96–1.02	0.623	0.92	0.86–0.98	0.009
BMI		0.99	0.97–1.02	0.438	1.01	0.98–1.04	0.723	0.95	0.91–1.00	0.059
Number of comorbidities		1.03	0.99–1.08	0.136	1.05	0.98–1.11	0.153	1.02	0.96–1.08	0.578
FVC		0.98	0.98–0.99	<0.001	0.99	0.98–0.99	0.002	0.98	0.97–0.99	0.004
GAP index										
	Stage I	1.00			1.00			1.00		
	Stage II	1.91	1.34–2.74	<0.001	2.11	1.40–3.18	<0.001	1.83	0.81–4.15	0.149
	Stage III	3.51	2.35–5.23	<0.001	4.13	2.60–6.57	<0.001	1.28	0.45–3.62	0.644
Physician's global assessment of clinical course of IPF										
	Stable disease	1.00			1.00			1.00		
	Slow progression	1.38	1.08–1.76	0.009	1.23	0.90–1.69	0.192	1.61	1.10–2.34	0.014
	Rapid progression	1.76	1.22–2.55	0.002	1.48	0.95–2.30	0.081	2.34	1.20–4.54	0.012
Antifibrotic therapy		0.74	0.60–0.90	0.004	0.76	0.59–0.99	0.049	0.71	0.51–1.98	0.043

*IPF, idiopathic pulmonary fibrosis; BMI, body mass index; FVC, forced vital capacity; GAP, gender-age-physiology index; HRCT, high resolution computed tomography; NYHA, New York Heart Association*.

When only considering elderly patients, in contrast to the entire cohort, GAP stage II and III were no significant predictors but the duration since first IPF symptoms (HR 0.92; 95%-CI 0.91–0.99; 0.009). Analyzing only nonelderly patients, slow progression and rapid progression were no significant predictors for mortality.

## Discussion

Under clinical practice conditions, derived from our data from the INSIGHTS IPF registry, over one third of IPF patients are 75 years or older at registry enrollment. Our study identified marked clinical differences between elderly patients and nonelderly patients with IPF including a higher number of comorbidities and reduced HRQoL. At baseline, patients received equally often antifibrotic therapy without differences in the effectiveness of therapy among age groups. All-cause mortality and unknown reasons for death were more often seen in elderly IPF. Antifibrotic therapy was a significant predictor for a better survival in both elderly and nonelderly patients.

Although our study showed that a significant number of patients with IPF are 75 years or older, data about this patient group and its clinical characterization are sparse. It is known that pulmonary function and oxygenation are declining with aging (17). While our elderly patients with IPF had a better FVC and FEV1 at baseline, the gas exchange and oxygenation were more impaired in comparison to nonelderly patients. This is in contrast to a study with a smaller sample size which identified no differences in lung function, gas exchange and oxygenation between patients with IPF ≥70 years and <70 years (18). Interestingly, duration since first IPF symptoms was shorter in elderly patients. Possible explanations are that, on the one hand, elderly patients have more comorbidities, which might lead to more frequent physician consultations with subsequent medical work-up and diagnosis of lung disease. On the other hand, time to diagnosis might be longer in nonelderly patients since differential diagnosis are more likely and diagnostic work-up is more thorough compared to elderly patients. Most likely due to risk assessment, less elderly patients underwent surgical lung biopsy and the diagnosis of IPF was more often based on HRCT in elderly patients. In our study, the number of comorbidities was higher in elderly IPF, mostly driven by a higher prevalence of cardio-vascular diseases such as left heart disease, coronary artery disease, peripheral arterial disease, atrial fibrillation and arterial hypertension. IPF is known to be associated with various cardiac comorbidities (6, 19, 20). Further, all of the comorbidities we identified are known to be more prevalent in an aging population (21, 22). The prevalence of cardiovascular comorbidities (i.e., coronary artery disease and arterial hypertension) was not only high in elderly, but also in nonelderly patients with IPF. It has been shown before, that arterial hypertension can be found in 14–71% of patients with IPF (6). The prevalence of coronary artery disease was reported to range between 4 and 68% in patients with IPF (6), with higher numbers in studies including patients on the waiting list for transplantation (23). The majority of patients in the INSIGHTS IPF registry have a history of smoking (10). Given the association between smoking and cardiovascular disease (24), this might partially explain the higher prevalence of coronary artery disease and arterial hypertension. There is also data showing that there is an association between patients with pulmonary fibrosis and coronary artery disease (25). Depression and anxiety are also frequently observed in patients with IPF, as patients with chronic lung disease are prone to psychological distress. In patients with ILD, depression and anxiety were found to be present in up to 23–49% and in 31%, respectively (26, 27). While in these studies with a smaller sample size, depression was independent from age, we found depression and anxiety to be less present in elderly IPF (26, 27). Perhaps elderly patients can cope better with the diagnosis of a fatal disease than nonelderly patients. Hence, comorbidities have to be acknowledged individually in the management of elderly patients with IPF. Overall, HRQoL was more reduced in elderly patients. The SGRQ, which was originally designed for COPD, showed no differences between age groups, which is in line with another study which identified the SGRQ independent from age in IPF (28). However, it must be noted that the EQ-5D and the WHO-5 used in our study are not IPF- or ILD-specific questionnaires. Still, we have shown recently in patients from the INSIGHTS IPF registry, that EQ-5D VAS and WHO-5 are associated with IPF disease progression as both parameters decrease significantly over time indicating a worsening HRQoL and patients with a decrease of >10% FVC over time have lower EQ-5D scores in the follow-up (14). Further, associations were seen between lower EQ-5D VAS and WHO-5 scores and mortality and also hospitalization (14).

A major focus of our study was antifibrotic therapy in an elderly IPF cohort. Recently, an US registry study identified age being negatively associated with antifibrotic therapy use (pirfenidone and nintedanib) (29). In contrast, in our study, more than half of elderly and nonelderly patients were treated with antifibrotic therapy at the time of enrollment, with no differences in age groups. Of note, there were significant differences concerning the drug distribution between age groups: while pirfenidone was more often used in nonelderly, nintedanib was more common in elderly IPF which might be explained by different side effect profiles and tolerability of the individual drugs. Recently, it has been shown that over 1 year follow up patients with IPF ≥75 years are more likely to discontinue pirfenidone and have a higher incidence of gastrointestinal disorders (30). In our study, dose reduction and discontinuation of therapy occurred equally often in both age groups. Lack of tolerability was more often a reason for discontinuation in elderly patients, while nonelderly discontinued more often due to efficacy failure or other reasons. Further, in our study, the course of FVC under antifibrotic therapy was independent from age groups, which is in line with data from a subgroup analysis of the two INPULSIS trials (nintedanib vs. placebo) (8). Taken together, this points out that the two antifibrotic therapies, which are currently the only approved medical treatment option in IPF (31), are effective in elderly patients and IPF patients shall therefore be treated with an antifibrotic therapy (32), regardless of the patients' age.

Another finding of our study was that all-cause mortality and death with unknown reasons were higher among elderly patients. This is in line with previous findings, where age has been identified as risk factor for mortality in IPF (12, 33). Interestingly, death related to IPF was not increased in elderly patients. In the present study, we identified several risk factors for early death in the entire cohort including the age group ≥75 years and no antifibrotic therapy. In 2018, data from the European IPF registry (eurIPFreg) suggested that patients with IPF and antifibrotic therapy have a better survival in comparison to patients without antifibrotic therapy (34). Recently, a positive effect of antifibrotic therapy on all-cause mortality was also identified using a large U.S. insurance data base without differences between pirfenidone and nintedanib (35). In line with these results, we also described a significantly better survival in IPF patients with antifibrotic therapy in the here analyzed INSIGHTS-IPF registry (16). In the present analysis of INSIGHTS-IPF, which is based on a considerably larger cohort and a longer follow-up, we could show that in elderly and nonelderly patients, antifibrotic therapy was significantly associated with better survival, which underlies again the benefit of antifibrotic therapy in patients with IPF independent from age. Beyond common predictors of mortality, there were also differences between the age groups. For example, duration since first IPF symptoms was a predictor for mortality, which was only identified in the elderly age group, but neither in the entire nor in the nonelderly age group. Further, slow and rapid progressions were significant predictors in elderly patients with IPF but not in nonelderly which underlines the importance of clinical assessment in the prognosis of disease in elderly patients. Interestingly, GAP stages II and III were no predictors for survival in elderly patients with IPF. This could be potentially caused by the fact, that all patients were ≥75 years, which qualifies every patient for the maximum of points in the “age” domain and leaves less discrimination (12). But more studies are needed to evaluate the usefulness of the GAP index in elderly patients.

Our study has strengths and limitations. The main strength is the large cohort of eligible patients; further strengths are the real-world setting, the prospective and multicentre data acquisition, and the comparatively long follow-up period. As limitations, first, owing to the (non-interventional) study time, the data collection varies in different study sites including documented visits and visit time-points depending on clinical routine schedules. Second, data were collected in highly experiences sites (possible selection bias). Third, transplantation was not included in the survival analysis since none of the elderly patients would have qualified for this intervention. Another limitation might be, that less elderly patients underwent surgical lung biopsy and the histological confirmation of an UIP pattern would strengthen the diagnostic confidence of IPF. However, over two thirds of elderly (and nonelderly) patients without surgical lung biopsy had UIP pattern on the HRCT scan. According to the guidelines, in patients with newly detected ILD with clinically suspected IPF and a HRCT pattern of UIP surgical lung biopsies is not essential (1, 36). In addition, the diagnosis of IPF was based on a multidisciplinary discussion in the majority of elderly and nonelderly patients, respectively. Further, a significant amount of data is missing in this retrospective analysis. The incompleteness of data especially affected the evaluation of HRQoL questionnaires, which were not available for all patients and the lung function over time under antifibrotic therapy. We could only include a limited number of patients in the longitudinal analysis of antifibrotic therapy effects, as we only included patients in whom an antifibrotic therapy was newly initiated and a sufficient follow-up was present. Further, we only included patients in this analysis if the antifibrotic therapy was started not earlier than 20 days before and not later than 20 days after a specific study visit, as described before (16). Using 20 days, it can be assumed that the FVC value will not be too strongly influenced by the onset of the therapeutic effect after the start of therapy. Choosing only 15 days as inclusion criteria would have clearly reduced the number of cases resulting in less robust results. Nevertheless, to our knowledge, this is the first study analyzing antifibrotic therapy in elderly patients longitudinally. The analyses were focused on the comparison of elderly and nonelderly patients including the outcomes of antifibrotic therapy. The comparison of patients treated and not treated with antifibrotic therapy was not adjusted by a propensity score. However, the association between antifibrotic treatment and mortality was analyzed in a multivariable Cox regression model including the parameters that were used to estimate the propensity score in Behr et al. resulting in comparable results (16). Concerning HRQoL questionnaires, currently, there are IPF-specific HRQoL questionnaires available such as an adaption of the SGRQ (37) or the Kings' Brief Interstitial Lung Disease health status (K-BILD) (38), but these were not included in the INSIGHTS-IPF registry. Finally, the cut-off of 75 years may seem arbitrary. Still, similar results in comorbidities, HRQoL, the effects of antifibrotic therapy and survival were seen when using a cut-off of 80 years in our study cohort, but due to a more limited number of patients and less robust data we decided to use the cut-off of 75 years for elderly vs. nonelderly patients. Further, the cut-off of 75 years has been used for the definition of “elderly” patients before (39).

In conclusion, a significant proportion of IPF patients are ≥75 years old and the management of these elderly patients requires consideration of the general health situation of elderly people. This includes more comorbidities, global reduced HRQoL and a higher all-cause mortality in elderly in comparison to nonelderly patients with IPF. The effects of an antifibrotic therapy do not differ between elderly and nonelderly patients with IPF and no antifibrotic therapy is a significant predictor for mortality in both age groups, emphasizing the importance for an early antifibrotic therapy in IPF, independent from age.

## Data Availability Statement

The datasets presented in this article are not readily available because the data will only be available upon reasonable request. Requests to access the datasets should be directed to Gabriela Leuschner, gabriela.leuschner@med.uni-muenchen.de.

## Ethics Statement

The studies involving human participants were reviewed and approved by Ethics Committee of the Medical faculty, Technical University, Dresden. The patients/participants provided their written informed consent to participate in this study.

## Author Contributions

GL, JK, JB, and NK analyzed and interpreted the data. MK, DP, AP, JK, HW, and JB were study steering committee members and contributed to the design and/or analysis of the data. All authors were involved in collecting the data, in writing the manuscript, and approved the final manuscript.

## Conflict of Interest

GL reports travel expenses/research grants/personal fees from Astra Zeneca, Boehringer Ingelheim, Novartis, Astra Zeneca, Forum Sanitos, and Roche/InterMune outside the submitted work. MK reports grants and personal fees from Roche/InterMune and Boehringer Ingelheim, outside the submitted work. AP reports grants and personal fees from Roche/InterMune and Boehringer Ingelheim, outside the submitted work. HW reports personal fees from Roche and Boehringer Ingelheim, outside the submitted work. DP has received fees for consultations outside the submitted work from Actelion, Biogen, Aspen, Amgen, Bayer, Daiichi Sankyo, and Sanofi. JB reports grants and personal fees from Boehringer Ingelheim, personal fees from Actelion, Roche, Galapagos, Promedior, BMS and MSD, during the conduct of the study. NK reports personal fees from Roche and Boehringer Ingelheim, outside the submitted work. The remaining authors declare that the research was conducted in the absence of any commercial or financial relationships that could be construed as a potential conflict of interest.

## Abbreviations

CI: confidence interval
COPD: chronic obstructive pulmonary disease
DLCO: diffusing capacity of the lung for carbon monoxide
EQ-5D: EuroQol five-dimensional questionnaire
FEV1: forced expiratory volume in 1
FVC: forced vital capacity
GAP: gender-age-physiology index
HR: hazard ratio
HRQoL: health-related quality of life
ILD: interstitial lung disease
IPF: idiopathic pulmonary fibrosis
SD: standard deviation
SGRQ: St. George's Respiratory Questionnaire
UCSD SOB: University of California San Diego Shortness of Breath Questionnaire
WHO-5: World Health Organization-5 Well-Being Index
6MWD: six-minute walk distance.
